# Impact of Intrinsic and Extrinsic Gaming Elements on Online Purchase Intention

**DOI:** 10.3389/fpsyg.2022.885619

**Published:** 2022-06-09

**Authors:** Xiaomei Wu, Silvia Santana

**Affiliations:** School of Economics and Management, Xiamen University of Technology, Xiamen, China

**Keywords:** intrinsic elements, extrinsic elements, gamification, purchase intention, S-O-R model, regulatory focus theory

## Abstract

Gamification is a developing trend that can work on customers' motivation and performance in online business areas. Notwithstanding, it is still vigorously debated as there is a continuous conversation inside the gamification community about whether individual gamification elements may really weaken or improve on customers' intrinsic and extrinsic motivations as well as the effect on the consumer's perceived enjoyment and purchase intention. The study uses a questionnaire survey as the research method. A total of 310 questionnaires were distributed, and after the data screening, 302 sets were valid data. The data analysis for this study was analyzed by using SPSS and Smart-PLS. The findings of this study show that intrinsic and extrinsic gaming elements affect consumers' purchase intention in gamification. This study shows how extrinsic gaming elements such as points, badges, feedback and challenges are affecting customers' perceived enjoyment. Furthermore, intrinsic gaming elements such as leaderboards, levels, avatars, and privacy control are affecting customers' perceived enjoyment. It also shows that perceived enjoyment positively affects purchase intention and mediates the relationship between intrinsic and extrinsic gaming elements and purchase intention. Additionally, it shows that promotion focus negatively moderates the relationship between intrinsic elements and perceived enjoyment. This study presents a new research model to explore the effect of extrinsic and intrinsic elements in gamification on purchase intention. The results of this research may help game designers to identify the right design features for the right customers, which has important practical implications for online business development.

## Introduction

As internet shopping has developed and become a basic channel for retailers, researchers have been expanding their attention around this area. Consumer experience has become a significant component of progress in contemporary retailing, expecting firms to look beyond pricing strategies and product innovation (Rose et al., 2011). In response to the market tension and developing rivalry, companies are compelled to search for new ways and strategies to attract the consideration of customers and connect with them in ways that form a long-term relationship with the company. Gamification, which can be defined as using game design elements and mechanics in nongame contexts (Deterding et al., 2011a), is such a new way. Over the last decade, gamification has been applied in several applications across diverse areas including web- technology and information systems (IS) (Aini et al., 2019, 2020). The most commonly used gaming elements are point, badge, and leaderboard. For example, Starbucks' membership reward program allows customers to accumulate stars through shopping, and the number of stars is related to gifts and membership levels, thereby increasing purchases and improving brand loyalty.

In spite of the fact that there are numerous different names for this idea (Deterding et al., 2011b), gamification is the main term that has been used to figure out how to sink into the industry and scholarly language. Gamification facilitates the intrinsic motivation of consumers (Domínguez et al., 2013), increases participation (Von Ahn and Dabbish, 2008; Witt et al., 2011), creates a better experience for consumers (Flatla et al., 2011; Gnauk et al., 2012), and increases brand connections (Berger et al., 2018), brand engagement (Xi and Hamari, 2020), digital sales (Eisingerich et al., 2019), hedonic and utilitarian values (Hsu and Chen, 2018), and product adoption (MüllerStewens et al., 2017).

Although there has been a lot of research on gamification marketing, there is a paucity of research on gamification about purchase intention and behavior, especially from the perspective of categorizing gaming elements by intrinsic and extrinsic motivations.

Psychologists have proposed various perspectives about motivation, including taking a glance at whether motivation emerges from the outside (extrinsic) or inside (intrinsic) of a person. Motivation as a significant focal point of this study is at the core of self-determination theory, which is divided into intrinsic and extrinsic (Deci et al., 2001). Autonomy, competence, and relatedness are three psychological necessities that are connected with intrinsic motivation. Intrinsic motivation gives autonomy to a person and provides him/her an opportunity to choose (Deci et al., 2001). When autonomy is diminished, it can diminish the innovativeness and performance and can likewise decrease the further desirability of the given sustainable activity (Gagné and Deci, 2005).

Current studies present the idea of motivation connected with gamification in two ways, namely, intrinsic and extrinsic (Wen et al., 2014). The blend of intrinsic and extrinsic motivations is significant for gamification achievement. In gamification, extrinsic motivation is connected with game elements like points and badges. Enjoyment, social acceptance, self-actualization, and recognition are connected with intrinsic motivation (Ryan and Deci, 2000; Gagné and Deci, 2005). Extrinsic motivation is the point at which consumers are persuaded to conduct a type of behavior or participate in an action since they need to acquire a prize or stay away from punishment (Tranquillo and Stecker, 2016). Intrinsic motivation is the point at which consumers takes part in a behavior since they think that it is fulfilling. They are acting for the sake of their wellbeing rather than for the sake of some external remuneration. The actual conduct is its own prize. Meaningful gamification focuses on the enjoyment of gaming, addresses the intrinsic motivation of an individual, and leads to the consumers' engagement and satisfaction (Deci et al., 1999; Schell, 2008; Ryan, 2012). Zichermann and Cunningham (2011) recommended that both intrinsic and extrinsic motivations should be considered in gamification.

The stimulus-organism-response (S-O-R) model (Mehrabian and Russell, 1974) has been to a great extent applied to explain consumers online purchase intention behaviors in the past literature (Liu et al., 2013). Dissimilar to conventional incentive systems that are used to excite consumers' extrinsic motivations, gamification systems focus on providing fascinating and striking elements that endeavor to invigorate consumers intrinsic motivations and social engagement (Hamari et al., 2014; Suh and Wagner, 2017; Feng et al., 2020). With regard to e-commerce, gamification highlights that online shopping activities are a huge trigger of customers' internal organism, which further impacts their purchase intention and behavior. Therefore, in this study, we will look into the intrinsic and extrinsic impacts of gamification on purchase intention by using the S-O-R model and explore the moderating effect of regulatory focus as well. In the research model, intrinsic and extrinsic elements are external stimulus, perceived enjoyment is an organism, and purchase intention is the response to the stimulus.

## Theoretical Background

Despite the fact that there is no all-inclusive meaning of gamification, Deterding et al. (2011a) definition is generally acknowledged to allude to gamification as contextualizing a game plan outside its unique space.

The mechanics-dynamics-aesthetics model can be used to clarify game plans according to a methodical perspective (Hunicke et al., 2004). It isolates game frameworks by breaking them into three distinct parts, namely, mechanics, dynamics, and aesthetics, which cooperate to make the utilitarian and hedonic values of the gameplay and impact the player's experience.

The gamification pyramid theory thinks that game elements contain game components, mechanics and dynamics. Game dynamics are at the top level, and elements are used to enhance consumer feelings and emotions. Game mechanics are the fundamental cycles that drive gamification and user commitment, such as contests and collaboration, investigations, asset securing, and so on. Game components are at the base; they contain points, badges, and leaderboards (Werbach and Hunter, 2012).

Xi and Hamari (2020) categorized game elements into immersion-related features, achievement-related features, and social interaction-related features. Immersion-related features basically attempt to submerge the player in independent, curious actions, including game mechanics such as avatars, narrating, account structures, and roleplay elements. Achievement-related features attempt to upgrade the players' feelings of achievement and incorporate game elements such as badges, challenges, missions, objectives, leaderboards, and progression metrics. Social interaction-related features are essentially used to empower consumers' social collaboration and incorporate game elements like group, gathering, and rivalry.

Xu et al. (2017) summarized the extrinsic and intrinsic gaming elements. Extrinsic gaming elements incorporate achievements, badges, rewards, gifting, feedback and reinforcement, pattern recognition, collecting, and so on, whereas intrinsic gaming elements include groups, messages, blogs, chat, progressive bars, levels, leaderboards, profiles, notification controls, avatars, privacy controls, and so on.

A number of authors suggest that badges, challenges, and leaderboards have the most impact on consumer behavior (Frith, 2012; Thom et al., 2012; Werbach and Hunter, 2012). Game elements, such as points and levels, have been and keep on being applied to an expansive range of nongame settings with shifting levels of achievement (Hamari et al., 2014; Seaborn and Fels, 2015). Instead of picking gamification elements in a vacuum, a superior way forward would be for scientists to use the crucial elements of game design (Schell, 2008). Researchers suggest both intrinsic and extrinsic elements that can motivate consumers should be considered to create a meaningful gamification experience. Those different elements may have different effects on consumer behavior. Werbach and Hunter (2012) examined that rewards might increase momentary action, while intrinsic motivation adds to long-haul commitment and enjoyment. Furthermore, the same element may have different effects. For example, Hamari (2013) mentioned that extrinsic motivation, for example, reward, has no impact on expanded playing activity. Zichermann and Cunningham (2011) pointed out that a few regularly used rewards, like cash, can de-spur the player. Thus, it is meaningful to explore how and to what extent these gaming elements have contributed to consumer motivation and behavior (Hamari et al., 2014; Seaborn and Fels, 2015).

## Research Model and Hypothesis Development

### Research Model

Being initiated in environmental psychology, the S-O-R system was created from the old-style stimulus-response theory. The S-O-R system comprises three fundamental components, namely, stimulus (external triggers that excite consumers' responses), organism (consumers' affective, cognitive, or normative evaluations of the external triggers), and response (consumers' behavioral outcomes of responses). In the context of e-commerce, gamification features (intrinsic and extrinsic elements) in the online shopping activities are huge triggers of consumers' perceived enjoyment, which further impacts their subsequent purchase intention. Correspondingly, this study considers gamification features as huge stimuli, perceived enjoyment as a noticeable organism, and purchase intention as the response in the research model.

**Figure 1:**
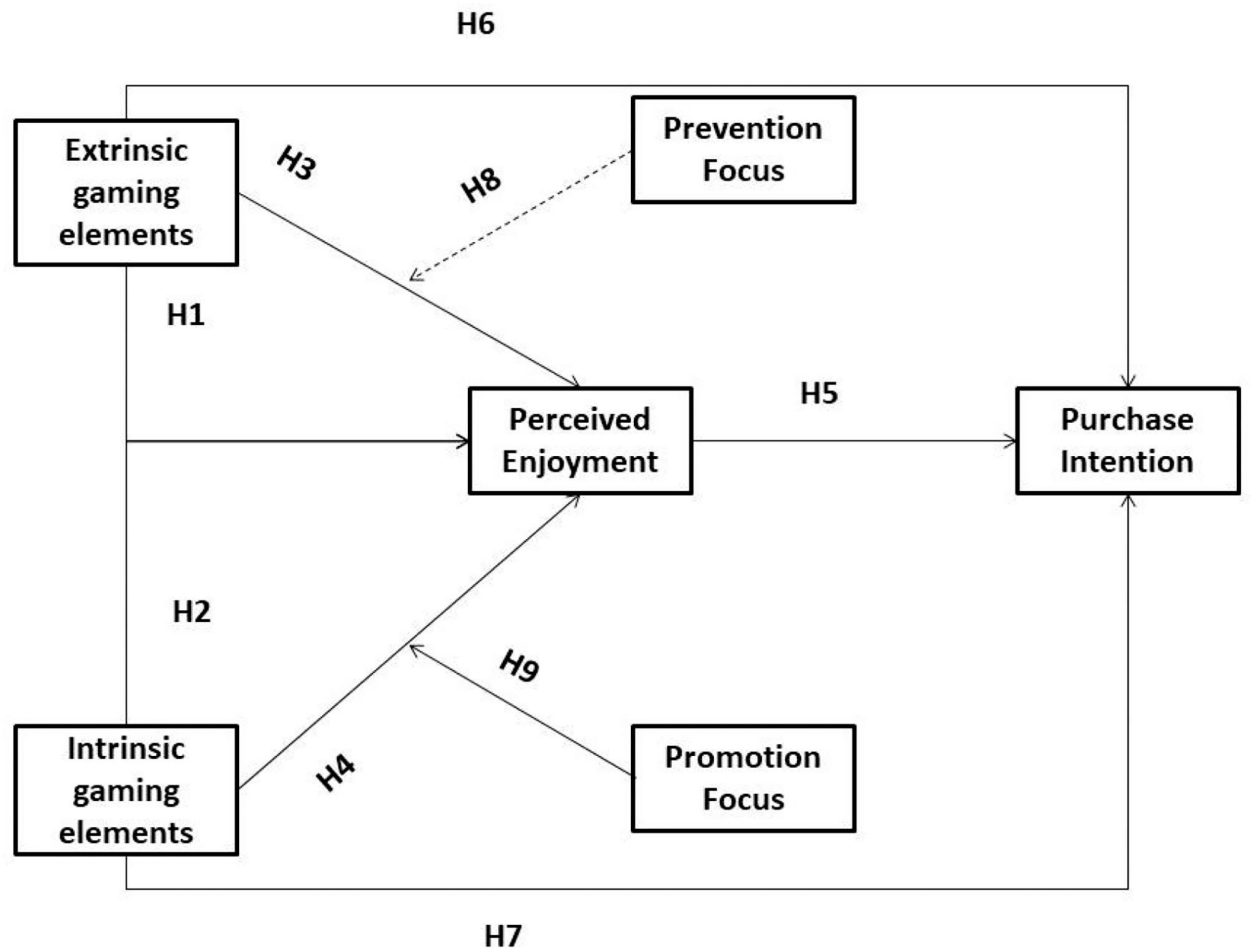
Research model.

The model underneath, which is displayed in Figure 1, includes two independent variables, namely, intrinsic and extrinsic gaming elements, one mediator, which is perceived enjoyment, one dependent variable, which is purchase intention, and two moderators, which are promotion focus and prevention focus.

### Hypothesis Development

#### The Relationship Between Extrinsic Gaming Elements and Purchase Intention

This study takes points, badges, feedback, and challenges for the extrinsic gaming elements (Xu et al., 2017), as these four elements are the most commonly used ones in gamification (Noah et al., 2017).

Points generally are a mathematical portrayal of compensating the player for activities completed in a game. Badges are the visual portrayal of an accomplishment, showing that the player has arrived at an explicit status or level (Werbach and Hunter, 2012). Feedback is furnishing the player with data about his performance in the game (Werbach and Hunter, 2012; Seaborn and Fels, 2015). A challenge is depicted as a drive expecting members to accomplish an assignment by defeating explicit obstructions. It empowers players to test their abilities (Zichermann and Cunningham, 2011).

Points and badges not only fill in as remunerations or stimuli for the shoppers but also rouse them to reconsider their behavior intention. Consumers gather points by partaking in explicit challenges on the gamified online shopping websites and achieving various targets (Sailer et al., 2014). The capacity to recover the award points and redo the virtual experience makes the consumers reconsider their intention to purchase (Tondello et al., 2017). Badges are used to increase consumer commitment levels and urge them to partake in various game-oriented tasks (Wang and Sun, 2011; Hamari, 2013). Several studies have already examined how game elements, such as points (Farzan et al., 2008) and badges (Denny, 2013; Hamari, 2013) affect consumer behavior.

According to Hamid and Kuppusamy (2017), hedonic elements, namely, feedback, progress, encouragement, achievement, and fun implementation are the core assistance of gamification applications to increase consumer motivation and support them to increase engagement.

During gamified shopping, purchase behavior is affected by challenges, for example, unlocking, empowering consumers to effectively defeat such challenges to unlock specific merchandise (Hildebrand et al., 2014). Consumers who take an interest in online business activities with amusement properties, such as games, are bound to make purchases (Feng et al., 2020).

Therefore, all of the four extrinsic elements are affecting the purchase intention. Thus, the study hypothesizes the following:

*H1: Extrinsic elements positively affect purchase intention*.

#### The Relationship Between Intrinsic Gaming Elements and Purchase Intention

This study takes leaderboard, level, avatar, and privacy control for the intrinsic gaming elements (Xu et al., 2017). First of all, Bittner and Shipper (2014) mentioned that purchase intention will be influenced by the intrinsic motivational incentives of game designs.

A leaderboard is a posting of consumers in light of their performance in the game. A level is a process for progressing in the game by gathering a specific number of points or completing explicit challenges (Werbach and Hunter, 2012; Seaborn and Fels, 2015). An avatar is a virtual portrayal of a consumer (Salam et al., 2014). Privacy control is a control that consumers must be able to set their own information secretly or openly (Francisco-Aparicio et al., 2013).

According to Noah et al. (2017), leaderboard and level are the commonly used gamification elements that could improve sensations of capability and accordingly increase intrinsic motivation and performance (Przybylski et al., 2010; Francisco-Aparicio et al., 2013). The utilization of an avatar leads to a more uplifting perspective toward the company and a more prominent purchase intention (Holzwarth et al., 2006). Individuals are worried about the privacy of their own information while making online purchases (Lorrie et al., 2000). Tsai et al. (2011) observed that, when open privacy data is available in search results, customers are willing to purchase from privacy-defensive websites, regardless of whether they are more costly. Thus, when there is an ability to control their privacy, the customers will be willing to purchase from the website/app. Therefore, all of the four intrinsic elements are affecting the purchase intention. Thus, the study hypothesizes the following:

*H2: Intrinsic elements positively affect purchase intention*.

#### The Relationship Between Extrinsic Gaming Elements and Perceived Enjoyment

The study by Kaynak and Basal (2019) found that extrinsic gaming elements positively influence perceived enjoyment. Previous studies recommended that customers have a higher level of enjoyment while collecting points and badges (Codish and Ravid, 2017). By achieving a specific assignment, consumers collects points, gets identification updates, and encounters a sensation of euphoria, fun, and enjoyment (Denny, 2013; Xi and Hamari, 2019). As indicated by Lazzaro (2009), there are four kinds of entertainment: easy fun, based on curiosity; serious fun, based on the excitement of obtaining valuable objects; people fun, based on social connections; and hard fun, based on challenges that require strategy and skill. Kapp (2012) mentioned that a system in which players engage in a challenge, defined by rules, interactivity, and feedback, that results in a quantifiable outcome often bring out an emotional response such as enjoyment. Research showed that players who got useful feedback following disappointment in the gamified environment communicated positive feelings about their experience (Herzig et al., 2015). Positive feelings incorporate joy, delight, excitement, and enjoyment; pessimistic feelings incorporate bitterness, disarray, and outrage (Laros and Steenkamp, 2005). Therefore, this study hypothesizes the following:

*H3: Extrinsic elements positively affect perceived enjoyment*.

#### The Relationship Between Intrinsic Gaming Elements and Perceived Enjoyment

A study by Koufaris et al. (2001) found that intrinsic gaming elements positively influence perceived enjoyment. The study has shown that comparisons can increase consumer's impression of capability and lead to increased enjoyment (Deci, 1971). High placement on a leaderboard is an attestation by the game that the player is competent on a given measure compared to other players and should lead to the feelings of competence. As sense of competence increases, enjoyment increases too. Wang et al. (2015) further observed that consumers performed best when given a difficult, but achievable, execution target (i.e., levels) rather than a moderate one. Avatar is a vital element of player recognizable processes as individuals are bound to relate to virtual characters that they see as more like themselves (Van Looy et al., 2012). Subsequently, playing and interfacing with avatars will increase enjoyment. Vorderer et al. (2004) found that if the individual can relate to the character about him or her, the individual will then look on the progression and consequences of the character's activities as critical to himself. Additionally, the user's feeling of being there, or his/her self-presence, is likewise fundamental for enjoyment. Privacy control while shopping on the websites, similar to the perception of losing control, is connected positively with uneasiness and hazard and that implies less shopping enjoyment for customers with high privacy control (Hwang and Kim, 2007). Therefore, when the customers can have control over their privacy, it will lead to an enjoyable feeling. Hwang and Kim (2007) contend that website quality with administration substance, which incorporates giving appropriate privacy data to the customers, positively affects customers' perceived enjoyment. Therefore, the study hypothesizes the following:

*H4: Intrinsic elements positively affect perceived enjoyment*.

#### The Relationship Between Perceived Enjoyment and Purchase Intention

A study by Raman (2020) found that perceived enjoyment positively influences purchase intention. Verhagen and Van Dolen (2011) mentioned that, to increase income and customer maintenance, online retailers should focus on perceived enjoyment. This infers that customers are more likely to enjoy in purchasing from the website when the method involved with shopping online is a pleasurable and agreeable experience (Cheema et al., 2013). Drawing upon the S-O-R model, perceived enjoyment was distinguished as a remarkable affective response to customers' purchase intention in the extant literature (Parboteeah et al., 2009). In particular, Chang and Chen (2015) uncovered that a higher hedonic perception (i.e., enjoyment) is useful to promote an online bidding imprudently. Enjoyment has been found to increase customers' intention to purchase on the website (Jiang and Benbasat, 2007; Floh and Madlberger, 2013). Therefore, the study hypothesizes the following:

*H5: Perceived enjoyment positively affects purchase intention*.

#### The Mediating Effect of Perceived Enjoyment

Taking into account that gamification elements are hedonistic and joy-arranged (Hassan and Hamari, 2019), the gamifying elements act as crucial motivators that impact a customer's perceived enjoyment (Aydin, 2018; Hassan and Hamari, 2019). Enjoyment and flow mediate the effect between motivational incentives and purchase intention (Bittner and Shipper, 2014). It has been adequately investigated in the past that perceived enjoyment helps in convincing the consumers to rehash their online purchasing behavior (Chiu et al., 2009). Additionally, customers highly value the idea of online shopping in view of the enjoyment quotient attached to it (Mathwick, 2002). Customers' purchase intention will be significantly improved when they see higher enjoyment in gamification. Based on the previous studies of H1-H5, this study hypothesizes the following:

*H6: Perceived enjoyment mediates the relationship between extrinsic elements and purchase intention*.
*H7: Perceived enjoyment mediates the relationship between intrinsic elements and purchase intention*.

#### The Moderating Effect of Regulatory Focus

As per the regulatory focus theory, promotion-focused people underline certain results and gains, while prevention-focused people accentuate pessimistic results and misfortunes (Shah et al., 1998). Self-regulatory focus is considered a significant source of differences in retail shopping behavior (Higgins et al., 1997; Arnold and Reynolds, 2009). Brockner and Higgins (2001) noted that “Regardless of whether individuals take on to a greater degree a promotion focus or prevention focus is a component of situational and dispositional factors.” Previous researches propose that regulatory focus is a significant determinant in individuals' data handling, assessment, and decision making (Dholakia et al., 2004). Promotion focus is related to attempting to achieve goals; prevention focus is related to completing obligations. Achieving a goal is something that many people feel that they need to do, that is, the behaviors that go with individuals' endeavors to achieve their desires are intrinsically motivated. Interestingly, completing obligations is something that the vast majority accept that they need to do. That is, the behaviors mirroring individuals' endeavors to satisfy their obligations are extrinsically motivated (Brockner and Higgins, 2001).

Regulatory focus orientations significantly moderated consumer shopping behavior (Das, 2015). This study is expected to use regulatory focus theory to represent how the gaming elements impact consumers' enjoyment of the online shopping website. Wan et al. (2011) demonstrated that a prevention-focused customer will in general see lower administrative quality when he/she observes a disappointment in service that happened to someone who is similar to him/her. Yang et al. (2013) likewise pointed out that a promotion focused customer will in general has more pessimistic feelings about a pre-process delay. Since promotion focus concerns about one's sensitivity to potential gains and rewards (Higgins et al., 1997; Brockner and Higgins, 2001), consumers with a promotion focus will not enjoy the process of using the gamified shopping website with intrinsic elements because they are focusing on improving themselves and will have a less enjoyable experience. Consumers with a high promotion focus have solid accomplishment and headway motivation. In particular, they give a lot of consideration to acclaims, awards, and improvements. When they did not get what they wanted, they would feel down and lack motivation. Conversely, an individual with a prevention focus will develop a self-protective reaction (such as avoiding any distractions). Thus, the prevention-focused consumers are likely to be more concentrated on the activities itself and have a more enjoyable experience while using the shopping website/app with extrinsic gaming elements. Therefore, the study hypothesizes the following:

*H8: The Prevention focus positively moderates the relationship between extrinsic elements and perceived enjoyment*.
*H9: The promotion focus negatively moderates the relationship between intrinsic elements and perceived enjoyment*.

## Research Design

### Measures

This study used the quantitative method, that is, a questionnaire survey as a research method. Purposive sampling was used in this study. English was used as the language in the questionnaires. The designed survey questionnaires included three sections: Section A (general questions), Section B (assessment of the variable items that are extrinsic elements, intrinsic elements, perceived enjoyment, purchase intention, and regulatory focus), and Section C (respondents' demographic profile). The questionnaire was distributed online using Google Form. The measurement scale of extrinsic gaming elements and intrinsic gaming elements are adapted from Schell (2008); Deterding et al. (2011a,b); McGonigal (2011); Zichermann and Cunningham (2011); perceived enjoyment was adapted from Yang et al. (2017) and Van der Heijden (2004); purchase intention was adapted from Bittner and Shipper (2014); prevention focus was adapted from Sheehan and Hoy (1999) and Lwin and Williams (2003); and promotion focus was adapted from Phelps et al. (2000). A 5-points Likert scale was used to measure all variables' elements in the measurement item: 1 = strongly disagree, 2 = disagree, 3 = neutral, 4 = agree, and 5 = strongly agree.

### Participants

The participants in this study are Indonesian customers who have experienced online shopping and gamification before. A pilot test of a total of 10 sets was distributed. Then, the questionnaire was distributed to 310 people in Indonesia in order to get the most reliable data. After the data screening, only a total of 302 sets are valid data.

As displayed in the demographic attributes of the respondents in Table 1, 47.4% women and 52.6% men. Most of the respondents are single (90.1%). The majority of the respondents are below 23 years old (48.3%). Most of the respondents are students (38.7%). The percentage of respondents with a monthly income of RMB 1,000 and below is 40.1%, of RMB 1,001–4,999 is 26.8%, of RMB 5,000–9,999 is 24.2%, and of RMB 10,000 and above is 8.9%. The percentage of respondents with secondary education is 30.1%, with a diploma is 14.6%, with a bachelor's degree is 52.3%, with a master's degree is 2.7%, and with PhD is 0.7%. Furthermore, there are 100% of respondents who used online shopping applications or websites before. Then, for the length of experience, 22.8% have used it for months to 2 years, 40.4% have used it 2–4 years, 8.6% have used it for 6–8 years, and 1.7% have used it for more than 8 years. The majority of them used Shopee (62.9%).

**Table 1:** Respondents' demographic profile.

	**Demographic**	**Frequency**	**Percentage (%)**
Gender	Men	143	47.4
	Women	159	52.6
Age	Below 23 years old	146	48.3
	23–33 years old	140	46.4
	34–44 years old	12	4.0
	45–55 years old	4	1.3
	Above 55 years old	0	0
Marital status	Single	272	90.1
	Married	30	9.9
Occupation	Student	117	38.7
	Teacher	2	0.7
	Government employee	5	1.7
	Company employee	113	37.4
	Businessman	38	12.6
	Unemployed	27	8.9
Monthly income	RMB 1,000 and below	121	40.1
	RMB 1,001–4,999	81	26.8
	RMB 5,000–9,999	73	24.2
	RMB 10,000 and above	27	8.9
Education	Secondary education	91	30.1
	Diploma	44	14.6
	Bachelor's degree	158	52.3
	Master's degree	7	2.7
	Doctoral (PhD)	2	0.7
Online shopping	Months to 2 years	69	22.8
Experience	2–4 years	122	40.4
	4–6 years	80	26.5
	6–8 years	26	8.6
	More than 8 years	5	1.7
Use most frequent	Aliexpress	2	0.7
	Lazada	89	29.5
	Shopee	190	62.9
	Tokopedia	6	2
	Others	15	5

## Data Analysis

The data was analyzed by using SPSS 24.0 and Smart-PLS 3.3.3. Kaiser Meyer Olkin (KMO) is 0.884, and Bartlett's test is 0.000. The common method variance (CMV) was 44.115%, which is above 20% and below 50%. Variance Inflated Factor (VIF) ought to be 5.0 or lower to ensure the model is free from multicollinearity issues (Hair et al., 2014). The Variance Inflated Factor (VIF) is lower than 5.0. The findings show that the various indices of model fit satisfy the recommended normalized values and indicate that the research model proposed in this exploration gives the best fit to the data collected (Fornell and Larcker, 1981).

### Validity and Reliability Analysis

Table 2 shows the result of reliability and convergent validity of the estimation model of the first order construct level. The reason why this study is having the indicator reliability quality evaluation is to conclude how much an indicator or a bunch of indicators was settled with what it intends to measure (Urbach and Ahlemann, 2010). The latent constructs of the loading indicator are suggested to be higher than 0.70 (Gefen et al., 2003). In contrast, the indicator of the loading factor, which is below 0.4, ought to be eliminated. This affirmed the reliability of indicators, in which all of the items' Cronbach's alpha are bigger than 0.7.

**Table 2:** Reliability and convergent validity.

	**Cronbach's alpha**	**Composite reliability**	**AVE**
Extrinsic (X1)	0.845	0.896	0.683
Intrinsic (X2)	0.895	0.927	0.761
Perceived enjoyment (M1)	0.817	0.892	0.734
Prevention focus (Z1)	0.720	0.842	0.640
Promotion focus (Z2)	0.705	0.834	0.627
Purchase intention (Y)	0.860	0.915	0.782

The composite reliability (CR) is used to quantify the items' dependability and to show good interior constancy, and the CR should be higher than 0.7 (Gefen et al., 2003). Table 2 shows the verification of the strong proof for the inside consistency measurement model of reliability of the composite reliability, which is higher than 0.7.

With the purpose to survey the correlation of indicators with its latent constructs, an appraisal of Average Variance Extracted (AVE) was completed. According to Hair et al. (2014), AVE reveals the degree to which a latent construct explains the distinction of its indicator. To accomplish satisfactory merged legitimacy, each construct needs to represent larger than 50% of the appointed indicators' variance (AVE > 0.50). Table 2 shows that all the constructs' AVE value is >50%.

In brief, all the Cronbach's alpha and CR are above 7.0. All of the AVE is above 0.50.

Discriminant validity discloses how many indicators separate crosswise over constructs or measure particular ideas by analyzing the relationships between proportions of possibly covering them. Discriminant validity was evaluated by the Fornell-Larcker criterion (Fornell and Larcker, 1981). It analyzes the square root of the AVE values with the latent variable relationships. Specifically, the square root of each construct's AVE should be greater than its highest correlation with any other construct. The rationale for the Fornell-Larcker criterion depends on the possibility that a construct imparts more differences to its related indicators than some other construct. Subsequently, Table 3 shows the evidence of the discriminant validity constructs' results.

**Table 3:** Discriminant validity.

	**Extrinsic**	**Intrinsic**	**Perceived**	**Prevention**	**Promotion**	**Purchase**
	**(X1)**	**(X2)**	**enjoyment (M1)**	**focus (Z1)**	**focus (Z2)**	**intention (Y)**
Extrinsic (X1)	0.827					
Intrinsic (X2)	0.554	0.872				
Perceived enjoyment (M1)	0.596	0.729	0.857			
Prevention focus (Z1)	0.507	0.454	0.485	0.800		
Promotion focus (Z2)	0.516	0.412	0.383	0.590	0.792	
Purchase intention (Y)	0.550	0.634	0.764	0.435	0.395	0.884

*The numbers in the diagonal are the square root of AVE, and others are the correlation coefficient of variables*.

### Testing of Hypothesis

The structural model covers the connection between the latent variables, which must be gotten from theoretical considerations. A structural model determines the way by which exogenous variables, such as path coefficient, coefficient of determinant (*R*^2^) and effect size (*F*^2^), in the model allude to hypothesis testing in the structural model assessment.

As Hair et al. (2014) mentioned, for the hypothesis to be accepted, *t*-value should be >2.33 and therefore, *p*-value should be <0.01, which implies that it is extremely significant, while hypothesis has been accepted in this study by assuming *t*-value > 1.645 and thus *p-*value <0.05, which implies that it is significant. Table 4 shows that extrinsic elements have a positive relationship with purchase intention (β = 0.122, *t*-value = 2.649, *p*-value = 0.008). H1 is supported. Intrinsic elements have a positive relationship with purchase intention (β = 0.134, *t*-value = 2.060, *p*-value = 0.040). H2 is supported. Extrinsic elements have positive relationship with perceived enjoyment (β = 0.237, *t*-value = 5.011, *p*-value = 0.000). H3 is supported. Intrinsic elements have positive relationship with perceived enjoyment (β = 0.441, *t*-value = 8.596, *p*-value = 0.000). H4 is supported. Perceived enjoyment has a positive relationship with purchase intention (β = 0.593, *t*-value = 8.616, *p*-value = 0.000). H5 is supported. Perceived enjoyment mediates the relationship between extrinsic elements and purchase intention (β = 0.141, *t*-value = 4.798, *p*-value = 0.000). H6 is supported. Perceived enjoyment mediates the relationship between intrinsic elements and purchase intention (β = 0.262, *t*-value = 5.890, *p*-value = 0.000). H7 is supported. The promotion focus does not moderate the relationship between extrinsic elements and perceived enjoyment (β = −0.019, *t*-value = 0.445, *p*-value = 0.657). H8 is not supported. The promotion focus negatively moderates the relationship between intrinsic elements and perceived enjoyment (β = −0.072, *t*-value = 2.432, *p*-value = 0.015). H9 is supported.

**Table 4:** Hypothesis testing.

	**Hypothesis**	**β**	***P*-Value**	***t*-value**	**Decision**	***F*** **^2^**
H1	Extrimsic -> PI	0.122^*^	0.008	2.649	Supported	0.023
H2	Intrinsic -> PI	0.134^*^	0.040	2.060	Supported	0.020
H3	Extrinsic -> PE	0.237^**^	0.000	5.011	Supported	0.083
H4	Intrinsic -> PE	0.441^**^	0.000	8.596	Supported	0.252
H5	PE -> PI	0.593^**^	0.000	8.616	Supported	0.370
H6	Extrinsic -> PE -> PI	0.141^**^	0.000	4.798	Supported	
H7	Intrinsic -> PE -> PI	0.262^**^	0.000	5.890	Supported	
H8	Extrinsic^*^Prevention Fcous -> PE	−0.019	0.657	0.445	Not supported	0.001
H9	Intrinsic^*^Promotion Focus -> PE	−0.072^*^	0.015	2.432	Supported	0.028

** *p < 0.01;*

* *p <0.05*.

*R*^2^ is a proportion of the model's prescient precision and it can likewise be seen as the consolidated impact of exogenous and endogenous factors. *R*^2^ speaks to the measure of change in endogenous constructs clarified by every single exogenous construct connected to it. There are three distinct guidelines of thumbs for adequate *R*^2^. As per Hair et al. (2014), the worthy value will be 0.75 as a significant value, 0.50 as a moderate value, and 0.25 as a powerless value. The result of *R*^2^ in this study is 0.621 for perceived enjoyment, which is a moderate value, and 0.605 for purchase intention, which is also a moderate value.

Effect side (*F*^2^) is an estimation used to survey the relative effect of an indicator contruct on an endogenous construct (Cohen, 1998). It evaluates how emphatically one exogenous construct adds to clarify a specific endogenous regarding *R*^2^. Impact estimate alludes to the distinction of *R*^2^ values with and without the predecessor construct. As indicated by Cohen (1998), the impact size of 0.35 is impressive from a substantial effect side, 0.15 is the medium effect side, and 0.02 is less effect side. The result of the *F*^2^ is in the range of 0.020–0.370, which means that some of the relationships have a small effect side and some have a substantial effect side. Furthermore, there is only one that is 0.001, which is the prevention focus as a moderating effect between extrinsic and perceived enjoyment, for which this hypothesis is not supported.

In total, eight out of nine hypotheses were supported. H1, H2, H3, H4, H5, H6, and H7 affirm that extrinsic and intrinsic gaming elements of gamification influence perceived enjoyment and purchase intention. H9 specifies that promotion focus negatively moderates the relationship between intrinsic gaming elements and perceived enjoyment. Finally, H8 shows that prevention focus does not moderate the relationship between extrinsic gaming elements and perceived enjoyment.

## Conclusion and Discussion

### Conclusion

This study plans to investigate what intrinsic and extrinsic gaming elements mean for customer's online purchase intention. Every one of the hypotheses except for H8 is supported.

First, the study shows that extrinsic gaming elements, namely, points, badges, feedback, and challenges, and intrinsic gaming elements, namely, leaderboards, levels, avatars and privacy control, positively influence the purchase intention. This result is consistent with the argument that gamification has a positive impact on the purchase intention of consumers who access mobile commerce platforms (Yu and Huang, 2022). This is also similar to Bittner and Shipper's (2014) statement that gamification affects purchase intention, but the degree of influence varies with prior gaming experience.

Second, the findings additionally affirm that extrinsic gaming elements and intrinsic gaming elements positively affect the perceived enjoyment. In the previous marketing literature, some studies have explored the effect of individual gaming elements on enjoyment. Game rewards, absorption, and autonomy of gamification positively enhance the sense of enjoyment (Xu et al., 2020). Positive challenges are positively related to online shopping enjoyment (Koufaris et al., 2001).

Third, perceived enjoyment was found to positively influence purchase intention. The result is consistent with the viewpoint that gamification elements (points, badges, and leaderboards) on mobile commerce platforms are key drivers that influence consumers' perceived pleasure (Hassan and Hamari, 2019).

Fourth, perceived enjoyment was found to mediate the relationship between extrinsic and intrinsic gaming elements and purchase intention. Previous literature resulted in enjoyment and flow as significant mediators between motivational incentives and purchase intention (Bittner and Shipper, 2014).

Additionally, the finding shows that promotion focus negatively moderates the relationship between intrinsic elements and perceived enjoyment. It indicated that, when customers with a promotion focus are participating in the gamification activities, they are too focused on the self-improvement, which then result in a less enjoyable shopping experience. Therefore, it also means that the promotion focus as a moderator reduces the feeling of enjoyment when interacting with the intrinsic gaming elements. In the context of gamification, when the consumers are focusing on the promotion, it makes them feel uncomfortable and burdened.

Finally, it was found that prevention focus does not moderate the relationship between extrinsic gaming elements and perceived enjoyment. Thus, H8 is not supported. As gamification is still new to the online shopping website/app, most of consumers have not faced any problems when using it, which means that the consumers have not considered that there is a need to prevent anything or avoid something negative while using the online shopping website.

### Research Contribution and Practical Implication

The study proposes important academic contributions in three ways. First, it applied the self-determination theory to explore the effects of gamification from an extrinsic and intrinsic motivation perspective. This study considered points, badges, feedback, and challenges for the extrinsic gaming elements that motivate consumers from the outside and leaderboard, level, avatar, and privacy control for the intrinsic gaming elements that motivate consumer from the inside (Xu et al., 2017). Most of the previous studies analyzed gamification based on intrinsic motivation (Bittner and Shipper, 2014; Mekler et al., 2015; Chan et al., 2018). The study extended gamification theory by showing the different effects of different gaming elements. The brand new perspective and research model will improve the gamification research and practice.

Second, the study employed the S-O-R model to study the influencing mechanism of gaming elements toward purchase intention. Perceived enjoyment plays an important mediating role between gaming elements and purchase intention, which enriches the application of the S-O-R model and makes the influencing mechanism clear and meaningful.

Third, this study examined the boundary conditions of the effect of gaming elements on perceived enjoyment. It took into account a consumer's individual characteristics. It focused on whether or not the promotion or prevention focus would have a moderating effect. This study found out that consumers with different regulatory foci would have different responses to the same stimulus. Studies about regulatory focus on gamification are still very rare, especially in terms of customers in e-commerce. Thus, it will help future researchers understand more and use it as a reference.

Finally, the study has meaningful practical implications. The research results may help the marketers to identify the right gamification design features for the right customers, which has important practical implications for online business development. It is a clue for game designers who are trying to influence customers' purchase intention through gamification. The game designers will know which gaming elements they should focus on to lead to positive emotion and enjoyment. The study will pave the way for better gamified applications.

### Limitation and Future Research

First of all, the extrinsic and intrinsic elements are only limited to four elements, thus future studies could include more of the elements to gain greater and richer research. Investigating more gamification elements would be beneficial in better understanding the effects of gamification. Next, as there is no relationship between the extrinsic elements and perceived enjoyment with prevention focus as the moderator, future research may explore other possible moderators. Furthermore, this research was done in Indonesia, an eastern country with emerging e-commerce. Future research can be carried out in different countries to see whether cultural differences or development levels make sense. Then, this research used a purposive sampling method, which may not reflect the entire population of consumers in the research area. Future research may use other sampling methods to get more valid and reliable data. Finally, experimental research methods can be used to explore the causality of gamification and other variables.

## Data Availability Statement

The raw data supporting the conclusions of this article will be made available by the authors, without undue reservation.

## Ethics Statement

Ethical review and approval was not required for the study on human participants in accordance with the local legislation and institutional requirements. The patients/participants provided their written informed consent to participate in this study.

## Author Contributions

XW contributed to the ideas generation, research guideline, and writing revision. SS contributed to the research design, data collection and analysis, and writing. All authors contributed to the article and approved the submitted version.

## Funding

Ministry of Education of China Humanities and Social Sciences Research Planning Fund Project provided funds for Research on the Effect of Gamification on Online Impulse Buying Behavior (Grant No. 17YJA630112).

## Conflict of Interest

The authors declare that the research was conducted in the absence of any commercial or financial relationships that could be construed as a potential conflict of interest.

## Publisher's Note

All claims expressed in this article are solely those of the authors and do not necessarily represent those of their affiliated organizations, or those of the publisher, the editors and the reviewers. Any product that may be evaluated in this article, or claim that may be made by its manufacturer, is not guaranteed or endorsed by the publisher.
