# Supplementary material for: Impact of Intrinsic and Extrinsic Gaming Elements on Online Purchase Intention
Source: Front Psychol. 2022 Jun 9;13:885619. doi: 10.3389/fpsyg.2022.885619 (PMC9220799; doi:10.3389/fpsyg.2022.885619)
Supplement: Supplementary file 1 [file Table_1.pdf]

Appendix

Construct	Measurement Items	References	Factor Loadings	t-values
**Extrinsic Elements**				
Feedback	The performance feedback I receive from the online shopping apps/ websites is helpful.	Cunningham and Zichermann, 2011; Deterding et al., 2011	0.808	22.813
Badges	The badges that can be obtained from the online shopping apps/ websites reflect the good work I have done		0.841	36.767
Challenges	The challenges and content in the online shopping apps/websites made me feel motivated.		0.801	24.788
Points	The points system correctly reflects my efforts when using the online shopping apps/websites.		0.855	41.756
**Intrinsic Elements**				
Avatars	My avatar in the online shopping apps/websites provides me with a kind of self-expression.	Schell, 2008; McGonigal, 2011	0.868	23.502
Privacy Control	Consumer control of personal information lies at the heart of consumer privacy.		0.864	31.478
Leaderboard	The reputation that I have as a user can be easily checked.		0.872	33.088

Levels	A level-up mechanism is essential to encourage my continued use of an online shopping apps/websites.		0.885	41.948
**Perceived Enjoyment**	The online shopping apps/websites was interesting.	Yang et al., 2017;	0.803	20.142
	The online shopping apps/websites made me feel enjoyable.	Van der Heijden, 2004	0.911	53.536
	The online shopping apps/websites was exciting.		0.853	28.918
**Purchase Intention**	I would purchase a product from the online shopping apps/websites.	Bittner and Shipper, 2014	0.896	45.951
	I intend to increase my online shopping activities in the future from this online shopping apps/websites.		0.912	60.460
	I intend to continue purchasing a product from this online shopping apps/websites in the future		0.844	19.818
**Prevention Focus**	I elect not to receive mail and phone solicitations from the online shopping apps/websites.	Sheehan and Hoy, 1999; Lwin and Williams, 2003	0.788	20.585
	I will set my server level e-mail filter to discard e-mails from this online shopping apps/websites.		0.836	24.529
	I will disguise my identity to prevent this online shopping apps/websites from finding me in future.		0.774	23.055
**Promotion Focus**	I will continue to update my personal information on this online shopping apps/websites database.	Phelps et al., 2000	0.863	16.089

I will inform this online shopping apps/websites of changes in my personal information.	0.754	14.273
I am willing to volunteer additional information to this online shopping apps/websites.	0.753	10.770
